# Prognostic Value of Postinduction Chemotherapy Volumetric PET/CT Parameters for Stage IIIA or IIIB Non–Small Cell Lung Cancer Patients Receiving Definitive Chemoradiotherapy

**DOI:** 10.2967/jnumed.120.260646

**Published:** 2021-12

**Authors:** Maja Guberina, Christoph Pöttgen, Martin Metzenmacher, Marcel Wiesweg, Martin Schuler, Clemens Aigner, Till Ploenes, Lale Umutlu, Thomas Gauler, Kaid Darwiche, Georgios Stamatis, Dirk Theegarten, Hubertus Hautzel, Walter Jentzen, Nika Guberina, Ken Herrmann, Wilfried E.E. Eberhardt, Martin Stuschke

**Affiliations:** 1 Department for Radiotherapy, West German Cancer Center, University Hospital Essen, University Duisburg–Essen, Essen, Germany;; 2 Department of Medical Oncology, West German Cancer Center, University Hospital Essen, University Duisburg–Essen, Essen, Germany;; 3 Division of Thoracic Oncology, West German Cancer Center, University Medicine Essen–Ruhrlandklinik, University Duisburg–Essen, Essen, Germany;; 4 German Cancer Consortium, Partner Site University Hospital Essen, Essen;; 5 Department of Thoracic Surgery and Thoracic Endoscopy, West German Lung Center, University Medicine Essen–Ruhrlandklinik, University Duisburg–Essen, Essen, Germany;; 6 Institute of Diagnostic and Interventional Radiology and Neuroradiology, University Hospital Essen, University Duisburg–Essen, Essen, Germany;; 7 Section of Interventional Pneumology, Department of Pulmonary Medicine, West German Cancer Center, University Medicine Essen–Ruhrlandklinik, University Duisburg–Essen, Essen, Germany;; 8 Institute of Pathology, West German Cancer Center, University Hospital Essen, University Duisburg–Essen, Essen, Germany; and; 9 Department for Nuclear Medicine, West German Cancer Center, University Hospital Essen, University Duisburg–Essen, Essen, Germany

**Keywords:** interim PET/CT, definitive radiotherapy, induction chemotherapy, stage III NSCLC, prognostic value

## Abstract

The aim of this follow-up analysis of the ESPATUE phase 3 trial was to explore the prognostic value of postinduction chemotherapy PET metrics in patients with stage III non–small cell lung cancer who were assigned to receive definitive chemoradiotherapy. **Methods:** All eligible stage IIIA (cN2) and stage IIIB patients in the trial received an induction doublet chemotherapy consisting of 3 cycles with cisplatin and paclitaxel, and subsequent combined chemoradiotherapy with a cumulative dose of up to 45 Gy (1.5 Gy per fraction twice a day), followed by a radiation boost (2 Gy once per day) with concurrent continuation of doublet chemotherapy with cisplatin and vinorelbine. The protocol definition prescribed a total dose of 65–71 Gy. ^18^F-FDG PET/CT was performed at study entry and before concurrent chemoradiotherapy. Interim PET metrics and known prognostic clinical parameters were correlated in uni- and multivariable survival analyses. Leave-one-out cross-validation was used to show internal validity. **Results:** Ninety-two patients who underwent ^18^F-FDG PET/CT after induction chemotherapy were enrolled. Median posttreatment MTV was 5.9 cm^3^. Altogether, 85 patients completed the whole chemoradiation with the planned total dose of 60–71 Gy. In univariable proportional-hazards analysis, each of 3 parameters—posttreatment MTV, posttreatment SUV_max_, and posttreatment maximum total lesion glycolysis (TLG_max(post)_)—was associated with overall survival (*P* < 0.05). Multivariable survival analysis, including clinical and postinduction PET parameters, found TLG_max(post)_ (hazard ratio, 1.032 [95% CI, 1.013–1.052] per 100 cm^3^ increase) and total radiation dose (hazard ratio, 0.930 [95% CI, 0.902–0.959] per 1 Gy increase) was significantly related to overall survival in the whole group of patients and in patients receiving a total dose of at least 60 Gy. The best leave-one-out cross-validated 2-parameter classifier was TLG_max(post)_ and total radiation dose. TLG_max(post)_ was associated with time to distant metastases (*P* = 0.0018), and posttreatment SUV_max_ was associated with time to locoregional relapse (*P* = 0.039), in multivariable analysis of patients receiving a total dose of at least 60 Gy. **Conclusion:** Postinduction chemotherapy PET parameters demonstrated prognostic significance. Therefore, interim ^18^F-FDG PET/CT is a promising diagnostic modality for guiding individualized treatment intensification.

## Visual Abstract

**Figure:**
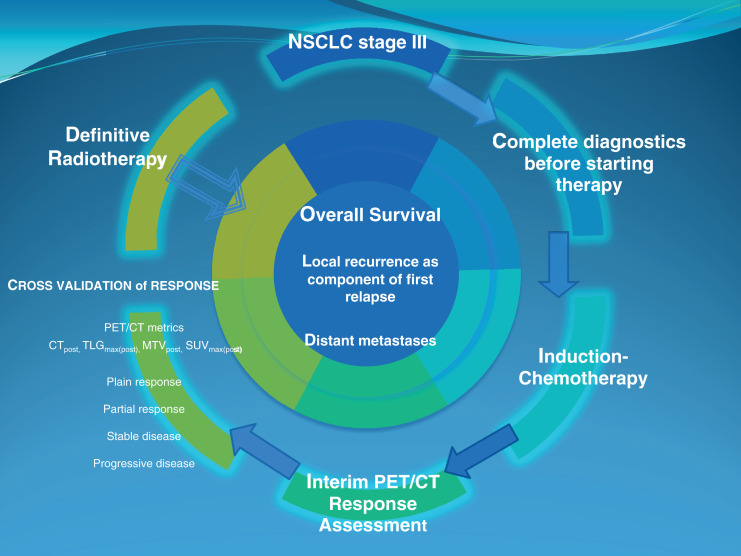

Induction chemotherapy followed by concurrent chemoradiotherapy is an important commonly applied variant of current regimes of definitive chemoradiotherapy for stage III non–small cell lung cancer (NSCLC). In the recent PACIFIC trial, about one fourth of the included patients with locally advanced stage III NSCLC received induction chemotherapy followed by concurrent chemoradiation and consolidation with durvalumab (*1*).

In addition, a significant survival benefit was found in metaanalyses on trials comparing surgery with induction chemotherapy and surgery for patients with operable stage III NSCLC (*2*,*3*). Prognostic factors to guide treatment intensification are needed, as progression-free survival at 18 mo of stage III NSCLC is less than 50% after concurrent chemoradiotherapy, even after durvalumab consolidation (*1*). Randomized and early phase II trials on radiation dose escalation were conducted for residual metabolic target volumes based on midradiation ^18^F-FDG PET/CT. Up to now, the first results have shown the feasibility of that approach and promising local tumor control (*4*,*5*). Such treatment pathways require that the metabolic tumor volume (MTV) on midtreatment PET/CT be a prognostic factor. Limited evidence is available that MTV or parameters based on MTV, such as total lesion glycolysis (TLG), are prognostic for stage III NSCLC patients treated with definitive chemoradiotherapy after induction chemotherapy. Ganem et al. assessed the influence of postinduction chemotherapy TLG on progression-free survival in 50 stage II or III NSCLC patients and concluded that postinduction ^18^F-FDG PET metrics might add value to estimate patients’ prognosis (*6*). Soussan et al. also found TLG to have prognostic value in 33 stage III NSCLC patients receiving induction chemotherapy and surgery or definitive radio- or chemoradiotherapy (*7*).

Prognostic value for pretreatment MTV (MTV_pre_) was not supported by our previous study on patients treated with induction chemotherapy followed by definitive chemoradiotherapy (*8*). This therapeutic regimen was particularly effective in patients with a high tumor burden and extensive initial MTV_pre_ disease (*8*).

However, the volume-based PET parameters posttreatment MTV (MTV_post_) and posttreatment TLG after induction chemotherapy might unmask tumor resistance and might have prognostic value.

Hence, the aim of this resulting analysis of the ESPATUE prospective phase 3 trial (*9*) was to explore the prognostic value of MTV_post_ followed by definitive chemoradiotherapy in patients with stage IIIA or IIIB NSCLC.

## MATERIALS AND METHODS

This analysis was based on the large, randomized phase 3 trial ESPATUE (*9*). All patients who had pathologically proven stage IIIA or IIIB NSCLC (Union for International Cancer Control TNM classification, 6th edition) and had been enrolled in that multicenter trial were evaluated. The ethics committee of the medical faculty of the University Duisburg–Essen approved this succeeding analysis. The included patients were treated from 2004 to 2012 at the University Hospital Essen. This site recruited 60% of all patients of the phase 3 trial. PET/CT scans from other institutions were not considered because those institutions either recruited fewer than 4 patients in total or the patients’ PET/CT scans were no longer available for quantitative analysis. The treatment protocol comprised induction doublet chemotherapy consisting of 3 cycles of cisplatin, 50 mg/m^2^, on days 1 and 8; paclitaxel, 175 mg/m^2^, on day 1 every 21 d; followed by combined chemoradiotherapy of up to 45 Gy given as 1.5 Gy twice a day, and a subsequent radiation boost of 2 Gy once per day up to 65–71 Gy with concurrent cisplatin, 50 mg/m^2^, and vinorelbine, 20 mg/m^2^, on days 2 and 9.

All included patients underwent PET/CT for initial staging before or no longer than 9 d after enrollment and 3 wk after induction chemotherapy before the start of radiotherapy. Those patients who underwent a repeated ^18^F-FDG PET/CT examination after induction chemotherapy, and for whom definitive chemoradiotherapy was intended and began, were eligible for this study.

### Imaging

All patients underwent ^18^F-FDG PET/CT at two time points, t1 and t2. The first one was performed at initial diagnosis, and the second one after 3 cycles of induction chemotherapy (follow-up before concurrent chemoradiotherapy). The baseline and follow-up ^18^F-FDG PET/CT scans were both obtained at the same center. PET/CT was performed on a Biograph mCT or Biograph Duo device (Siemens). Details about the PET/CT acquisition and ^18^F-FDG administration have been reported previously (*10*).

To measure the MTV, this study used a combination of a hybrid-based approach, visual interpretation and delineation, and an automatic segmentation procedure analogous to the method used in the Radiation Therapy Oncology Group/American College of Radiology Imaging Network RTOG 1106/ACRIN6697 trial (*11*). The RTOG 1106 method uses a fixed source-to-background ratio in combination with CT anatomy-based manual editing to exclude mediastinal normal tissues. The initial and current CT morphology is mandatory information. For shrinking tumors, we limited the MTV_post_ to the initial MTV_pre_. Normal tissues such as large vessels, as well as tumor extent, were manually defined in a detailed examination done by two expert radiation–oncologists in consensus. The activity within the postinduction MTV_post_ was evaluated against the background of normal tissue. For the background definition, the mean activity in 1 mL of blood pool within the aortic arch was determined. MTV_post_ had to be above 1.5 times the background activity and was limited by the tumor borders on CT (*12*). For automatic contouring, the Eclipse treatment planning system, version 15.5, was used (Varian Medical Systems) (*13*–*15*). The MTV_post_ and maximal tracer activity value within the tumor volume (Bq/cm^3^; posttreatment SUV_max_ [SUV_max(post)_]) were measured. Posttreatment maximum total lesion glycolysis (TLG_max(post)_) was calculated as the product of MTV_post_ and SUV_max(post)_. Because the SUV_max_ and SUV_mean_ of the lesion strongly correlate with each other, with correlation coefficients above 0.93 in many studies, we did not determine SUV_mean_ (*16*–*18*).

### Statistics

The primary endpoints were overall survival, time to progression, and time to locoregional progression as a component of the first recurrence or distant progression alone.

Event times were defined as the interval between the patient’s entering the study and the time of the event. PET metrics combined with other known prognostic factors were analyzed for prediction of treatment outcome.

A prognostic *n*-parameter classifier (*n* = 1–4) was built from the parameters of the postinduction chemotherapy PET/CT. A score-selection method for proportional-hazards regression with leave-one-out cross-validation was applied (*13*,*19*). The leave-one-out cross-validation approach was performed using the SAS macro described by Rushing et al. (*20*). The variables for the best *n*-parameter model were selected on the basis of the highest χ^2^ score for the proportional-hazards model in comparison to all other *n*-parameter models. Classifiers were calibrated on a training dataset, leaving out the *i*th patient. The endpoint was overall survival. The *i*th patient was then classified as high-risk or low-risk depending on its predictive risk score according to the classifier from the training dataset. This procedure was repeated for each patient. Patients with a cross-validated predictive index greater than the median in the respective training dataset were classified as high-risk, and the other patients were classified as low-risk.

Kaplan–Meier survival curves for the high-risk and low-risk groups were compared using the nonparametric log-rank test. In addition, overall survival in the high-risk and low-risk groups was compared using the Cox proportional-hazards model, and the corresponding hazard ratios and their 95% CIs were reported.

As measures of relative interpatient heterogeneity, the classic coefficient of variation and the quartile coefficient of dispersion were used, the latter being calculated according to $\frac{Q_{3}-Q_{1}}{Q_{1}+Q_{3}}$, where *Q*_1_ and *Q*_3_ represent the first and third quartiles of the distribution, respectively (*21*).

The proportional-hazards analysis and receiver-operating-characteristic analysis were performed using the PHREG procedure in SAS/STAT statistical software, version 14.3 (*13*). The validity of the proportional-hazards assumption was assessed by a Kolmogorov-type supremum test (PHREG procedure; SAS).

## RESULTS

Altogether, 92 patients from the ESPATUE trial fulfilled the inclusion criteria of this study. All patients were enrolled at the University Hospital Essen from 2004 to 2012 (*9*,*10*). The median follow-up for the patients who were alive at the time of the study was 94.8 mo (range, 67.1–159.9 mo). Patient and tumor characteristics are shown in Table 1. The interim PET/CT at t2 was performed within a median of 8 d (10th–90th percentiles, 5–15 d) before the start of radiotherapy. In total, 62 patients relapsed. Among them, 32 had a locoregional relapse as a component of the first recurrence and 30 relapsed at distant sites alone. There was a significantly positive Spearman correlation coefficient between MTV_post_ and SUV_max(post)_ (0.74; *P* < 0.0001), between MTV_post_ and TLG_max(post)_ (0.98; *P* < 0.0001), and between SUV_max(post)_ and TLG_max(post)_ (0.83; *P* < 0.0001). TLG_max(post)_ revealed the greatest intertumor heterogeneity of the parameters, characterized by a coefficient of variation of 370.5% and a quartile coefficient of dispersion of 94.7%, followed by MTV_post_, with a coefficient of variation of 245.5% and a quartile coefficient of dispersion of 87.4% (Table 1). For comparison, the intertumor heterogeneity of TLG_max(pre)_ and MTV_pre_ was smaller in pretreatment PET/CT, with a coefficient of variation of 164.2% and 109.8%, respectively, and a quartile coefficient of dispersion of 63.0% and 57.2%, respectively. In an analysis of the ratios between TLG_max(post)_ and MTV_post_ and their respective initial values on the first PET/CT exam, the variations were also smaller according to their coefficients of variation, 185.2% and 117.1%, respectively, than those of the respective parameters from the interim PET/CT.

**TABLE 1:** Patient and Tumor Characteristics for All Patients Who Started with Definitive Chemoradiotherapy (*n* = 92)

Characteristic	Data
Sex (*n*)	
Female	22
Male	70
ECOG performance status (*n*)	
0	57
1	34
2	1
Age (y)	
Median	58.5
Range	41.0–74.0
Tumor category	
cT1–2	28
cT3	7
cT4	57
Nodal category	
cN0–N1	32
cN2–N3	60
Histology	
Squamous cell carcinoma	39
Adenocarcinoma	36
Other	17
MTV_post_ (cm^3^)	
Median	5.9
Range	0.0–540.8
Interquartile range (Q1–Q3)	1.6–23.8
Coefficient of variation (%)	245.5
SUV_max(post)_	
Median	3.6
Range	<1.0–38.4
Interquartile range (Q1–Q3)	2.6–6.6
Coefficient of variation	99.9%
TLG_max(post)_	
Median	20.8
Range	0.0–10,058
Interquartile range (Q1–Q3)	3.5–128.9
Coefficient of variation (%)	370.5
Total radiation dose	
Median	71.0
Range	3.0–72
Interquartile range (Q1–Q3)	66.0–71.0

ECOG = Eastern Cooperative Oncology Group; Q1 and Q3 = first and third quartiles of distribution, respectively.

Univariable analysis of the postinduction chemotherapy PET/CT parameters using proportional-hazards analysis revealed that for all 3 parameters, MTV_post_, SUV_max(post)_, and TLG_max(post)_, the hazards ratios for an increase of 1 unit in the variables were greater than 1. The unit was 10 cm^3^ for MTV_post_, 100 cm^3^ for TLG_max(post)_, and 10 for SUV_max(post)_. The *P* values for the association with survival became smaller with increasing coefficients of variation of the parameters. No deviations from the proportional hazards or the functional form of the covariate were observed for any of these parameters (*P* > 0.05, Kolmogorov supremum test).

Multivariable proportional-hazards analysis of all covariates shown in Table 1 revealed that total radiation dose related positively and TLG_max(post)_ negatively to longer survival, using forward selection at an α value of 0.05. All patients were intended to receive a total dose of 65–71 Gy as per the protocol. Our per-protocol definition prescribes a total dose of at least 60 Gy. Seven patients received a total dose of less than 60 Gy. Two of these 7 died during radiotherapy. Because reasons for stopping the radiotherapy early were, or might be, directly related to survival, we also performed a second analysis to look for the effect of total dose on survival in the per-protocol group of 85 patients. Again, total radiation dose and TLG_max(post)_ remained significant prognostic factors for survival (Table 2). The internal validity of the prognostic value of the parameters from the postinduction chemotherapy PET/CT was assessed using leave-one-out cross-validation. The best 2- to 4-parameter signatures were consistently included and revealed that TLG_max(post)_ was best in 99% or more of the leave-one-out training dataset and is therefore the most important parameter from postinduction chemotherapy PET/CT to predict survival, provided the prescribed radiation dose is applied. The generalizability of the 2-parameter signature to the held-out training dataset was better than that of the 3- or 4-parameter signature as indicated by the lowest *P* value for the differences between cross-validated survival curves for the high- and low-risk groups at an α value of 0.05 (Table 3).

**TABLE 2:** Univariable and Multivariable Proportional-Hazards Analysis of Parameters from Postinduction Chemotherapy PET/CT Using Forward Parameter Selection at α Value of 0.05

Prognostic variable	Hazard ratio	95% CI	*P* (χ^2^ test)
Univariable survival analysis (all eligible patients who started with definitive chemoradiotherapy [*n* = 92])			
MTV_post_	1.042	1.007–1.079	0.017
SUV_max(post)_	1.486	1.062–2.080	0.043
TLG_max(post)_	1.028	1.009–1.048	0.0044
Multivariable survival analysis (all eligible patients [*n* = 92])			
Total radiation dose	0.930	0.902–0.959	<0.0001
TLG_max(post)_	1.032	1.013–1.052	0.0002
Multivariable survival analysis (all patients who received at least 60-Gy total dose [*n* = 85])			
Total radiation dose	0.891	0.813–0.977	0.0142
TLG_max(post)_	1.034	1.014–1.054	0.0008
Multivariable time to progression analysis (all patients who received at least 60-Gy total dose [*n* = 85])			
TLG_max(post)_	1.038	1.018–1.058	0.0001
Total radiation dose	0.848	0.770–0.934	0.0008
Multivariable time to distant progression alone analysis (all patients who received at least 60 Gy total dose [*n* = 85])			
TLG_max(post)_	1.037	1.014–1.061	0.0018
Multivariable time to locoregional progression as component of first relapse analysis (all patients who received at least 60-Gy total dose [*n* = 85])			
Total radiation dose	0.807	0.705–0.924	0.0019
cT3 tumor category	3.605	1.326–9.800	0.012
SUV_max(post)_	1.070	1.003–1.141	0.039

All clinical and PET parameters listed in Table 1 were included in analysis. Hazard ratios are given per 10 cm^3^ increase in MTV, or per SUV_max_ increase of 10, or per TLG increase of 100 cm^3^.

**TABLE 3:** Leave-One-Out Cross-Validated 1- to 4-Parameter Classifiers for Separating High-Risk from Low-Risk Groups by Median Predictive Index According to Parameters Shown in Table 1

		High-risk vs. low-risk groups		
Parameter	Percentages of LOO-CV loops	*P* (log-rank test)	Hazard ratio	95% CI
1-parameter best classifier (all 92 eligible patients who started with definitive chemoradiotherapy)				
Total dose	99	0.036	1.62	1.03–2.55
TLG_max(post)_	1			
1-parameter TLG_max(post)_ classifier (all 92 eligible patients who started with definitive chemoradiotherapy)	100	0.036	1.60	1.03–2.51
2-parameter best classifier (all 92 eligible patients)				
Total dose	100	0.0006	2.17	1.38–3.42
TLG_max(post)_	99			
3-parameter best classifier (all 92 eligible patients)				
Total dose	100	0.0069	1.84	1.17–2.89
TLG_max(post)_	99			
cT4	88			
4-parameter best classifier (all 92 eligible patients)				
Total dose	100	0.017	1.73	1.10–2.71
TLG_max(post)_	99			
cT4	95			
cN2/N3	93			
2-parameter best classifier (all 85 patients who received at least 60-Gy total dose)				
Total dose	100	0.0026	2.05	1.27–3.30
TLG_max(post)_	99			

Percentages of LOO-CV (leave-one-out cross-validation) loops indicate consistency with which parameter is selected into best *n* parameter model across all LOO iteration loops.

Endpoint is overall survival

**FIGURE 1.**
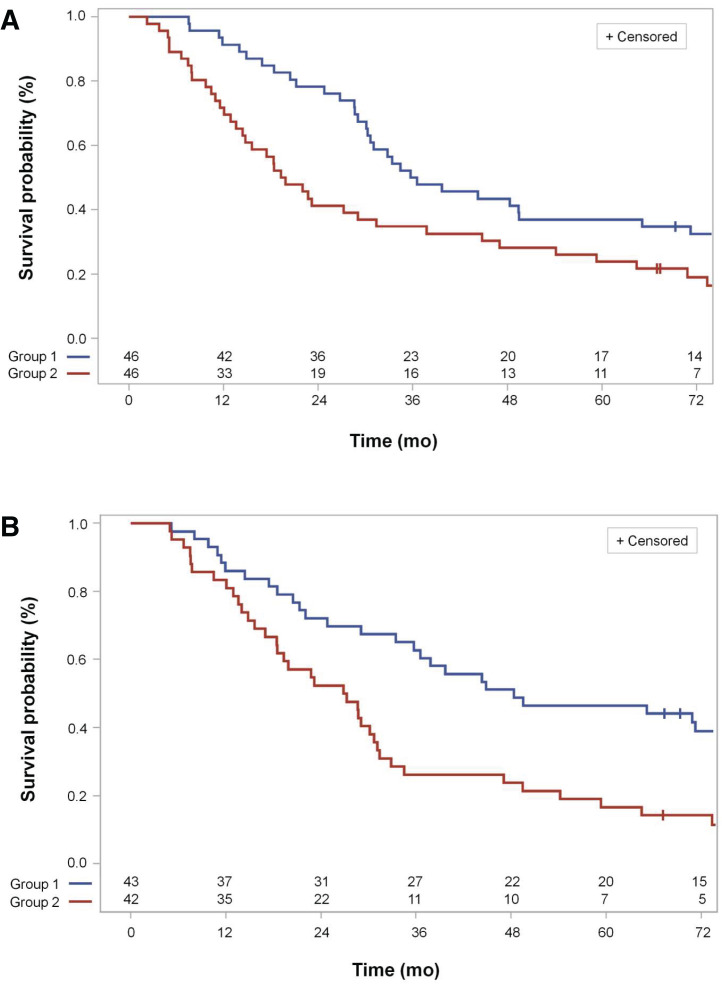
(A) Cross-validated Kaplan–Meier survival curves for high- and low-risk groups of all eligible patients according to median TLG_max(post)_ in training dataset (*P* = 0.036, log-rank test (*n* = 92), for differences between survival curves). Group 1, low-risk group, has TLG_max(post)_ below or at median of TLG_max(post)_ in respective leave-one-out training dataset. Group 2, high-risk group, has TLG_max(post)_ above median in respective leave-one-out training dataset. (B) Cross-validated Kaplan–Meier survival curves for patients who received chemoradiotherapy up to total dose of at least 60 Gy as per protocol according to best 2-parameter classifier. Best leave-one-out cross-validated 2-parameter classifier is best classifier contained in 99% of leave-one-out loop parameters total dose and TLG_max(post)_ and in 1% of leave-one-out loop parameters total dose and SUV_max(post)_ (*P* = 0.0026, log-rank test (*n* = 85), for differences between survival curves). Group 1, low-risk group, has linear predictor built from complementary leave-one-out training dataset below or at median of values in training dataset. Group 2, high-risk group, has linear predictor above median.

Figure 1A shows the split of the cross-validated survival curves in the high- and low-risk group of all included patients (*n* = 92), according to the observation that the TLG_max(post)_ of the respective leave-one-out observation was greater than the median of group 2 or less than or equal to the median of the training dataset (group 1).

Figure 1B shows the split of the survival curves for the 85 patients treated as per the protocol with a total radiation dose of at least 60 Gy, in accordance with the best 2-parameter classifier analysis consisting of total dose and TLG_max(post)_ in 99% and total dose and SUV_max(post)_ in 1% of the cross-validation loops (Table 3; Fig. 1B).

In an exploratory manner, we determined the optimal cut point for MTV_post_ and TLG_max(post)_ to divide patients into high-risk and low-risk groups by a time-dependent receiver-operating-characteristic analysis according to the criterion of maximum sum of sensitivity and specificity (Youden *J* statistic). For survival at 60 mo, the areas under the receiver-operating-characteristic curve for MTV_post_ and TLG_max(post)_ were 0.634 (95% CI, 0.528–0.7445) and 0.643 (95% CI, 0.532–0.755), respectively. The corresponding cut points for MTV_post_ and TLG_max(post)_ were 2.9 cm^3^ and 11.3, respectively. This is close to the cut point of maximum separation of the high-risk and low-risk groups according to the log-rank test (Supplemental Fig. 1; supplemental materials are available at http://jnm.snmjournals.org), which is at 2.3 cm^3^ for MTV_post_ and 9 for TLG_max(post)_. There is a second cut point for MTV_post_ and TLG_max(post)_ at higher values, 20 cm^3^ and 75, resulting in a second local maximum separation of the high-risk and low-risk groups at reversed group sizes. This emphasizes that MTV_post_ and TLG_max(post)_ are continuous risk parameters with a constant hazard ratio per unit increase. The respective survival curves, separated according to TLG_max(post)_ cut points of 11.3 and 75, are shown in Supplemental Fig. 1.

**FIGURE 2.**
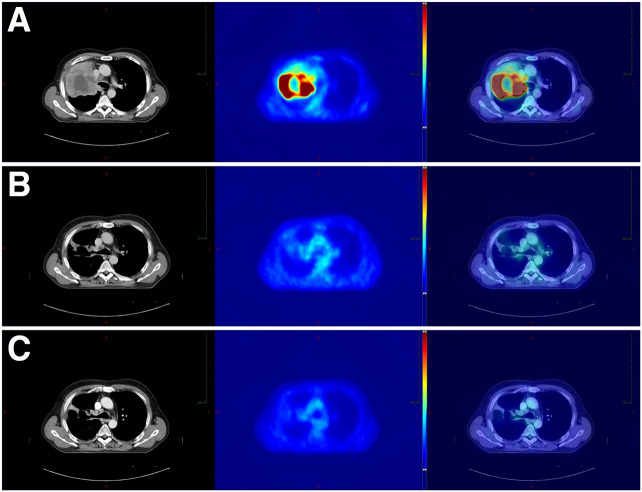
Initial (A), interim (B), and follow-up (C) ^18^F-FDG PET/CT in patient who had poorly differentiated NSCLC and complete remission after induction doublet chemotherapy with cisplatin and paclitaxel followed by definitive chemoradiation with cisplatin and vinorelbine and total dose of 71 Gy, with overall survival > 60 mo. Initial images (2A) show vivid ^18^F-FDG uptake before start of treatment (MTV_pre_, 480.1 cm^3^). Interim images (2B) show response after 3 cycles of chemotherapy (7 d before start of chemoradiation). There is complete PET response, with SUV_max(post)_ below 1.5 times background activity (central 1 cm^3^ of blood pool within aortic arch) in low-risk group that had MTV_post_ below defined cut point (MTV_post_, ≤2.9 cm^3^). Follow-up images (2C) show complete fading of metabolic activity after induction chemotherapy and completion of concurrent chemoradiation during follow-up (10.9 mo after start of radiotherapy).

Figure 2 shows a patient with complete remission of the central lung cancer on PET imaging and with a curative outcome after induction chemotherapy and definitive chemoradiation.

**FIGURE 3.**
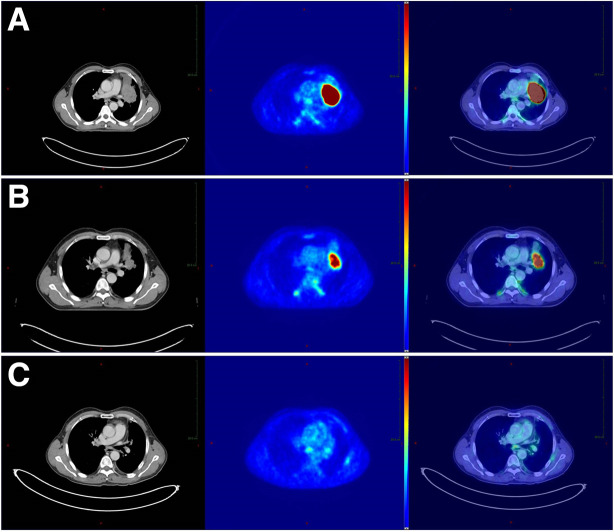
Initial (A), interim (B), and follow-up (C) ^18^F-FDG PET/CT in patient who had poorly differentiated NSCLC and partial remission after induction doublet chemotherapy with cisplatin and paclitaxel before definitive chemoradiation with cisplatin and vinorelbine and a total dose of 71 Gy, with overall survival of 21 mo. Initial images (3A) show vivid ^18^F-FDG uptake before start of treatment (MTV_pre_, 72.8 cm^3^). Interim images (3B) show residual ^18^F-FDG uptake after 3 cycles of chemotherapy before definitive chemoradiation in high-risk group that had MTV_post_ above defined cut point (MTV_post_, >2.9 cm^3^). The follow-up images (3C) at 10.5 mo after start of radiotherapy initially also show a very good tumor response, but unfortunately not persistent in the further course of the disease history. Progression in field started 12 mo after primary treatment. The latter may indicate that tumor heterogeneity and the most resistant subvolumes are of importance for local control after definitive chemoradiotherapy.

Because parameters related to MTV from postinduction PET/CT were significantly related to overall survival, we also analyzed the relation of the endpoint to the pattern of relapse, that is, time to progression, time to locoregional progression as a component of the first relapse analysis, and time to distant progression alone. TLG_max(post)_ was a significant prognostic factor for the time-to-progression and time–to–distant-metastasis endpoints using multivariable proportional-hazards analysis combined with forward selection (Table 2). For the time to locoregional progression as a component of the first relapse endpoint, SUV_max(post)_ was the single important factor from PET/CT.

## DISCUSSION

There is a current need to find a valid diagnostic procedure to predict therapy response in lung cancer. For systemic therapies such as chemotherapy or immune checkpoint blockade in lung cancer, PERCIST, version 1.0, proved to be a valuable tool to calculate treatment outcome (*22*,*23*).

In this ensuing analysis of a large, randomized phase 3 trial, we aimed to evaluate known prognostic clinical parameters combined with volumetric PET/CT metrics after induction chemotherapy. Complete data to long-term follow-up and sites of relapses are available in this study.

MTV_post_, SUV_max(post)_, and TLG_max(post)_ are associated with prognosis in univariable analysis for all stage III NSCLC patients of this trial who started with definitive chemoradiotherapy after induction chemotherapy and did not undergo surgery. TLG_max(post)_ was the most prognostic factor agglomerating the prognostic information of both MTV_post_ and SUV_max(post)_. We have previously shown that the percentage decrease in SUV_max_ obtained from the pre- and postinduction chemotherapy PET/CT has prognostic relevance in the entire group of randomized patients treated with definitive chemoradiotherapy or a trimodality therapy after induction chemotherapy (*10*), and we have demonstrated in this study that the postinduction chemotherapy PET/CT provides important prognostic information.

However, the MTV_pre_ obtained from the pretherapeutic PET/CT did not show prognostic value for patients intended for definitive chemoradiotherapy in this trial (*8*). Extending the analysis by MTV-based factors from the postinduction chemotherapy PET/CT on this cohort of patients who were not treated with surgery, the present results reinforce the prognostic value of the postinduction chemotherapy PET/CT (Figs. 1–*3*). From all patient-derived and PET-derived factors, TLG_max(post)_ demonstrated the greatest association with overall survival. This finding showed internal validity since TLG_max(post)_ was consistently selected as the best 2- or 4-parameter classifier in 99% of leave-one-out validation loops for the total radiation dose given.

In addition, we performed a subgroup analysis excluding patients who received a total radiation dose of less than 60 Gy, and again, the same parameters remained significant. Prospective trials did not find any important influence of total radiation dose on survival (*24*). In this trial, the radiation dose was escalated with dose volume limits for the entire lung, so that mean lung dose could not exceed 19 Gy. Because total dose was not randomized, there might be some hidden factors acting on both selection of the total radiation dose and prognosis. However, the analysis in this trial showed that the total dose remains an important factor, either in the per-protocol group of patients receiving a total dose of at least 60 Gy or together with the most significant tumor dependent factor, that is, TLG_max(post)_.

Studies revealing that MTV_post_ or TLG_max(post)_ can have a high prognostic value after induction chemotherapy originated from esophageal cancer (*25*,*26*) and squamous cell carcinomas of the head and neck region (*27*,*28*), but data from NSCLC are scant (*6*,*7*). This analysis considerably adds to evidence of the prognostic relevance of TLG_max(post)_ after induction chemotherapy for patients who were consecutively treated with definitive chemoradiotherapy.

With respect to the potential implications of therapy intensification based on PET prognostic parameters, the site of treatment failure is important. In the analysis of the additional endpoints “distant progression alone” and “locoregional progression” as a component of the first relapse, TLG_max(post)_ was more strongly related to distant progression whereas SUV_max(post)_ was more strongly related to locoregional progression, potentially indicating that tumor heterogeneity and the most resistant subvolumes are important in local control after definitive chemoradiotherapy (*29*). Whether a larger set of PET/CT-based radiomics features along with a deep-learning approach will result in better prognostication from postinduction chemotherapy PET/CT remains an open question for further studies (*30*).

There are some limitations in the interpretation of these results. This was not a confirmatory but an initial exploratory study. Interim PET/CT is not yet a standard diagnostic procedure. Potential ways to adapt treatment to a poor PET response in the future include delivering a dose-escalated boost to a residual MTV; intensifying concurrent chemoradiotherapy with, for example, simultaneous immune checkpoint inhibitors; or enhancing consolidation therapies. A further potential limitation of the applicability of these results is that the induction chemotherapy was performed before definitive chemoradiotherapy in only a minority of centers. Thus, 26.8% of patients in the PACIFIC trial received induction chemotherapy (*1*). However, there is currently great interest in combining induction chemotherapy followed by definitive chemoradiotherapy with immunotherapy and consolidation therapy with a checkpoint inhibitor (PACIFIC BRAZIL trial ClinicalTrials.gov identifier NCT04230408; ESPADURVA trial ClinicalTrials.gov identifier NCT04202809). The ESPADURVA trial compares induction chemotherapy and chemoradiotherapy followed by surgery or a radiotherapy boost with and without immunotherapy. The ADMIRAL trial (NCT04372927) avoids mediastinal irradiation in patients with a complete response after 3–4 cycles of chemoimmunotherapy and radiotherapy of the primary tumor. Validated response-dependent prognostic parameters during treatment are of high importance for such schedules of adaptive treatment intensification.

## CONCLUSION

Interim PET/CT after induction chemotherapy confers important prognostic information before definitive chemoradiotherapy in patients with locally advanced NSCLC and should be considered as standard for radiotherapy planning after induction chemotherapy.

## DISCLOSURE

The ESPATUE trial was supported by the German Cancer Aid under grant 70-3070-Eb. The West German Cancer Center at University Hospital Essen is supported by the Oncology Centre of Excellence Program of the German Cancer Aid (grant 110534) and by the German Federal and State governments via the German Cancer Consortium (DKTK). Thomas Gauler is on an advisory board or is a consultant for Ipsen, Novartis, BMS, and Eisai; receives honoraria from BMS, Ipsen, Novartis, MSD, Eisai, and Pfizer; receives traveling expenses from BMS, Ipsen, Novartis, MSD, Eisai, and Pfizer; and owns stocks in Bayer. Christoph Pöttgen receives honoraria from Roche Pharma and personal fees from Boehringer Ingelheim and Astra Zeneca. Ken Herrmann receives personal fees from Bayer, Sofie Biosciences, SIRTEX, Adacap, Curium, Endocyte, BTG, IPSEN, Siemens Healthineers, GE Healthcare, Amgen, Novartis, ymabs, Bain Capital, and MPM Capital. Wilfried Eberhardt receives honoraria as an advisory board function from Astra Zeneca, BMS, Roche, MSD, Pfizer, Boehringer, Takeda, Eli Lilly, Bayer, and Celgene; honoraria for educational lectures from BMS, MSD Astra Zeneca, Roche, Novartis, Pfizer, Boehringer, Takeda, Abbvie, Celgene, and Eli Lilly; and research grants from Eli Lilly, BMS, and Astra Zeneca. Martin Metzenmacher receives honoraria from BMS, Boehringer Ingelheim, MSD, Roche, and Takeda. Marcel Wiesweg receives honoraria from Amgen, Boehringer Ingelheim, Novartis, Roche, and Takeda and research funding from Bristol-Myers Squibb and Takeda. Martin Schuler is a compensated consultant for AstraZeneca, Boehringer Ingelheim, Bristol-Myers Squibb, Janssen, Novartis, Roche, and Takeda and receives honoraria for continuing medical education presentations from Amgen, Boehringer Ingelheim, Bristol-Myers Squibb, Janssen, MSD, and Novartis. His institution receives research funding from AstraZeneca, Boehringer Ingelheim, Bristol Myers-Squibb, and Novartis. Martin Stuschke receives honoraria as an advisory board function from AstraZeneca, Bristol-Myers Squibb, Sanofi-Aventis, and Janssen-Cilag. His institution received research funding from AstraZeneca in 2019 and 2020. No other potential conflict of interest relevant to this article was reported.Figure 3

**KEY POINTS**

**QUESTION:** Can postinduction PET metrics direct chemoradiation in locally advanced NSCLC?

**PERTINENT FINDINGS:** The results are based on the ESPATUE phase 3 trial. MTV_post_, SUV_max(post)_, and TLG_max(post)_, together with total radiation dose, have the power to predict local and systemic control, as well as overall survival.

**IMPLICATIONS FOR PATIENT CARE:** Interim PET/CT after induction chemotherapy confers important prognostic information before definitive chemoradiotherapy and is a promising diagnostic modality for guiding individualized treatment intensification for NSCLC patients being treated with curative intent.

## References

[1] Antonia SJ Villegas A Daniel D. Durvalumab after chemoradiotherapy in stage III non–small-cell lung cancer. N Engl J Med. 2017;377:1919–1929. 28885881 10.1056/NEJMoa1709937

[2] Song WA Zhou NK Wang W. Survival benefit of neoadjuvant chemotherapy in non-small cell lung cancer: an updated meta-analysis of 13 randomized control trials. J Thorac Oncol. 2010;5:510–516. 20107424 10.1097/JTO.0b013e3181cd3345

[3] Horita N Miyazawa N Morita S. Preoperative chemotherapy is effective for stage III resectable non–small-cell lung cancer: metaanalysis of 16 trials. Clin Lung Cancer. 2013;14:488–494. 23664722 10.1016/j.cllc.2013.03.006

[4] Kong FM Ten Haken RK Schipper M. Effect of midtreatment PET/CT-adapted radiation therapy with concurrent chemotherapy in patients with locally advanced non-small-cell lung cancer: a phase 2 clinical trial. JAMA Oncol. 2017;3:1358–1365. 28570742 10.1001/jamaoncol.2017.0982 PMC5674997

[5] Raman S Bissonnette JP Warner A. Rationale and protocol for a Canadian multicenter phase II randomized trial assessing selective metabolically adaptive radiation dose escalation in locally advanced non-small-cell lung cancer (NCT02788461). Clin Lung Cancer. 2018;19:e699–e703. 29903551 10.1016/j.cllc.2018.05.002

[6] Ganem J Thureau S Gouel P. Prognostic value of post-induction chemotherapy ^18^F-FDG PET-CT in stage II/III non-small cell lung cancer before (chemo-) radiation. PLoS One. 2019;14:e0222885. 31603916 10.1371/journal.pone.0222885 PMC6788704

[7] Soussan M Chouahnia K Maisonobe JA. Prognostic implications of volume-based measurements on FDG PET/CT in stage III non-small-cell lung cancer after induction chemotherapy. Eur J Nucl Med Mol Imaging. 2013;40:668–676. 23306807 10.1007/s00259-012-2321-7

[8] Guberina M Eberhardt W Stuschke M. Pretreatment metabolic tumour volume in stage IIIA/B non-small-cell lung cancer uncovers differences in effectiveness of definitive radiochemotherapy schedules: analysis of the ESPATUE randomized phase 3 trial. Eur J Nucl Med Mol Imaging. 2019;46:1439–1447. 30710323 10.1007/s00259-019-4270-x

[9] Eberhardt WE Pottgen C Gauler TC. Phase III study of surgery versus definitive concurrent chemoradiotherapy boost in patients with resectable stage IIIA(N2) and selected IIIB non-small-cell lung cancer after induction chemotherapy and concurrent chemoradiotherapy (ESPATUE). J Clin Oncol. 2015;33:4194–4201. 26527789 10.1200/JCO.2015.62.6812

[10] Pöttgen C Gauler T Bellendorf A. Standardized uptake decrease on [^18^F]-fluorodeoxyglucose positron emission tomography after neoadjuvant chemotherapy is a prognostic classifier for long-term outcome after multimodality treatment: secondary analysis of a randomized trial for resectable stage IIIA/B non-small-cell lung cancer. J Clin Oncol. 2016;34:2526–2533. 27247220 10.1200/JCO.2015.65.5167

[11] Mahasittiwat P Yuan S Xie C. Metabolic tumor volume on PET reduced more than gross tumor volume on CT during radiotherapy in patients with non-small cell lung cancer treated with 3DCRT or SBRT. J Radiat Oncol. 2013;2:191–202. 23795245 10.1007/s13566-013-0091-x PMC3686305

[12] RTOG 1106/ACRIN 6697: randomized phase II trial of individualized adaptive radiotherapy using during-treatment FDG-PET/CT and modern technology in locally advanced non-small cell lung cancer (NSCLC). American College of Radiology website. https://www.acr.org/-/media/ACR/NOINDEX/Research/ACRIN/Legacy-Trials/ACRIN-6697_RTOG1106.pdf. Published February 25, 2014. Accessed August 31, 2021.

[13] SAS Institute Inc. Chapter 87: The PHREG procedure. In: *SAS/STAT^®^ 14.3 User’s Guide.* SAS Institute Inc.; 2017:1–277.

[14] Cheebsumon P Boellaard R de Ruysscher D. Assessment of tumour size in PET/CT lung cancer studies: PET- and CT-based methods compared to pathology. EJNMMI Res. 2012;2:56. 23034289 10.1186/2191-219X-2-56 PMC3502476

[15] Erdi YE MO Larson SM Imbriaco M Yeung H Finn R Humm JL. Segmentation of lung lesion volume by adaptive positron emission tomography image thresholding. Cancer 1997;80(suppl):2505–2509. 9406703 10.1002/(sici)1097-0142(19971215)80:12+<2505::aid-cncr24>3.3.co;2-b

[16] Zhang J Chen L Chen Y. Tumor vascularity and glucose metabolism correlated in adenocarcinoma, but not in squamous cell carcinoma of the lung. PLoS One. 2014;9:e91649. 24614132 10.1371/journal.pone.0091649 PMC3948888

[17] Huang YE Chen CF Huang YJ Konda SD Appelbaum DE Pu Y. Interobserver variability among measurements of the maximum and mean standardized uptake values on ^18^F-FDG PET/CT and measurements of tumor size on diagnostic CT in patients with pulmonary tumors. Acta Radiol. 2010;51:782–788. 20707663 10.3109/02841851.2010.497772

[18] van Gómez López O García Vicente AM Honguero Martínez AF. Heterogeneity in [^18^F]fluorodeoxyglucose positron emission tomography/computed tomography of non-small cell lung carcinoma and its relationship to metabolic parameters and pathologic staging. Mol Imaging. 2014;13:1–12. 10.2310/7290.2014.00032 25248853

[19] Simon RM Subramanian J Li MC Menezes S. Using cross-validation to evaluate predictive accuracy of survival risk classifiers based on high-dimensional data. Brief Bioinform. 2011;12:203–214. 21324971 10.1093/bib/bbr001 PMC3105299

[20] Rushing C Bulusu A Hurwitz HI Nixon AB Pang H. A leave-one-out cross-validation SAS macro for the identification of markers associated with survival. Comput Biol Med. 2015;57:123–129. 25553357 10.1016/j.compbiomed.2014.11.015 PMC4306627

[21] Ospina R Marmolejo-Ramos F. Performance of some estimators of relative variability. Front Appl Math Stat. 2019;5:43.

[22] Beer L Hochmair M Haug AR. Comparison of RECIST, iRECIST, and PERCIST for the evaluation of response to PD-1/PD-L1 blockade therapy in patients with non-small cell lung cancer. Clin Nucl Med. 2019;44:535–543. 31021918 10.1097/RLU.0000000000002603

[23] Minamimoto R Takeda Y Hotta M. ^18^F-FDG and ^11^C-4DST PET/CT for evaluating response to platinum-based doublet chemotherapy in advanced non-small cell lung cancer: a prospective study. EJNMMI Res. 2019;9:4. 30649637 10.1186/s13550-019-0472-2 PMC6335230

[24] Bradley JD Paulus R Komaki R. Standard-dose versus high-dose conformal radiotherapy with concurrent and consolidation carboplatin plus paclitaxel with or without cetuximab for patients with stage IIIA or IIIB non-small-cell lung cancer (RTOG 0617): a randomised, two-by-two factorial phase 3 study. Lancet Oncol. 2015;16:187–199. 25601342 10.1016/S1470-2045(14)71207-0 PMC4419359

[25] Chhabra A Ong LT Kuk D. Prognostic significance of PET assessment of metabolic response to therapy in oesophageal squamous cell carcinoma. Br J Cancer. 2015;113:1658–1665. 26657654 10.1038/bjc.2015.416 PMC4702001

[26] van Rossum PSN Fried DV Zhang L. The value of ^18^F-FDG PET before and after induction chemotherapy for the early prediction of a poor pathologic response to subsequent preoperative chemoradiotherapy in oesophageal adenocarcinoma. Eur J Nucl Med Mol Imaging. 2017;44:71–80. 27511188 10.1007/s00259-016-3478-2 PMC5121174

[27] Kim KR Shim HJ Hwang JE. The role of interim FDG PET-CT after induction chemotherapy as a predictor of concurrent chemoradiotherapy efficacy and prognosis for head and neck cancer. Eur J Nucl Med Mol Imaging. 2018;45:170–178. 28940101 10.1007/s00259-017-3836-8 PMC5745569

[28] Rudžianskas V Korobeinikova E Rudžianskienė M. Use of ^18^F-FDG PET/CT imaging for radiotherapy target volume delineation after induction chemotherapy and for prognosis of locally advanced squamous cell carcinoma of the head and neck. Medicina (Kaunas). 2018;54:107. 10.3390/medicina54060107 PMC6306774 30544718

[29] Aerts HJ van Baardwijk AA Petit SF. Identification of residual metabolic-active areas within individual NSCLC tumours using a pre-radiotherapy ^18^fluorodeoxyglucose-PET-CT scan. Radiother Oncol. 2009;91:386–392. 19329207 10.1016/j.radonc.2009.03.006 PMC4693609

[30] Lou B Doken S Zhuang T. An image-based deep learning framework for individualizing radiotherapy dose. Lancet Digit Health. 2019;1:e136–e147. 31448366 10.1016/S2589-7500(19)30058-5 PMC6708276

